# Non-surgical Management of a Malecot Tube Migrating From a Small Bowel Fistula

**DOI:** 10.7759/cureus.58630

**Published:** 2024-04-20

**Authors:** Payton C O'Quinn, Lou M Smith, Alexander C Cavalea

**Affiliations:** 1 Department of Surgery, East Tennessee State University Quillen College of Medicine, Johnson City, USA; 2 Department of Surgery, University of Tennessee Medical Center, Knoxville, USA

**Keywords:** gastroenterology, wound care, retained foreign body, enterocutaneous fistula, critical care, trauma

## Abstract

Foreign bodies are encountered relatively often within the practice of general surgery. We present a unique case of a rubber, self-retaining, radiopaque “mushroom-tip” Malecot tube placed for fistula drainage control due to an enterocutaneous fistula (ECF) that became a gastrointestinal foreign body.  A 24-year-old male presented in shock with gunshot wounds to his right chest and right upper abdomen to a Level I trauma center. He required a prolonged hospital stay with additional urological and thoracic procedures and an interventional radiology procedure for hepatic pseudoaneurysm and subsequently developed an ECF. The patient was discharged to a rehabilitation facility with a wound management system (WMS) for ECF drainage but returned to the clinic with chemical burns and skin excoriation due to poorly controlled output and suboptimal WMS fit. A better fitting WMS was employed and a 20-French Malecot catheter was placed to assist with drainage control. The patient later returned with abdominal pain reporting the Malecot advanced forward spontaneously and was not externally visible. CT scan revealed the Malecot across the prior ileocolic anastomosis. After considering potential treatment options, we initially proceeded with aggressive bowel stimulation, and saline enemas hoping the tube would pass through his colostomy. He was discharged and the catheter passed at home a few days later via the stoma. Gastroenterological literature recommends invasive management for sharp, corrosive, or elongated foreign bodies exceeding 6cm in length. This unusual case demonstrates a 30-centimeter (cm) blunt object passing through the small bowel and colon in the absence of an ileocecal valve.

## Introduction

Retained and ingested foreign bodies are an issue of significant and not infrequent clinical concern in the field of surgery. These incidents can be harmful to patients and may necessitate surgical intervention. Foreign bodies are most commonly the result of surgical procedures, with an incidence of almost one for every 5,500 operations [1]. Most non-surgical foreign bodies in the adult population are ingested orally or entered intentionally via the anus. Foreign bodies often pass through an intact digestive system without endoscopic or surgical intervention, although 20% require endoscopic removal and 1% require surgical intervention [2]. Foreign bodies, regardless of location, have the possibility of causing serious clinical complications for the patient and have been associated with perforation, localized pain, and perforation [3]. Most adult patients will present in emergency departments with clinical symptoms. Ingested foreign bodies generally present differently than surgical foreign bodies and are thus managed differently. One example of how object characteristics influence management is that sharp ingested objects pose a greater risk of gastrointestinal tract perforation than blunt or round objects and thus typically necessitate endoscopic removal [4]. When managing foreign bodies retained within the gastrointestinal tract, special attention must be given to the anatomical location of the foreign body. The esophagus, pyloric channel, and if distal to the stomach, the ileocecal valve are locations where objects often become lodged [5]. Guidelines exist for such management but may not always provide definitive guidance. We present a unique case of a rubber, self-retaining radiopaque, “mushroom-tip” Malecot catheter placed in an enterocutaneous fistula (ECF) that migrated and was retained in the gastrointestinal tract as a foreign body.

## Case presentation

**Figure 1:**
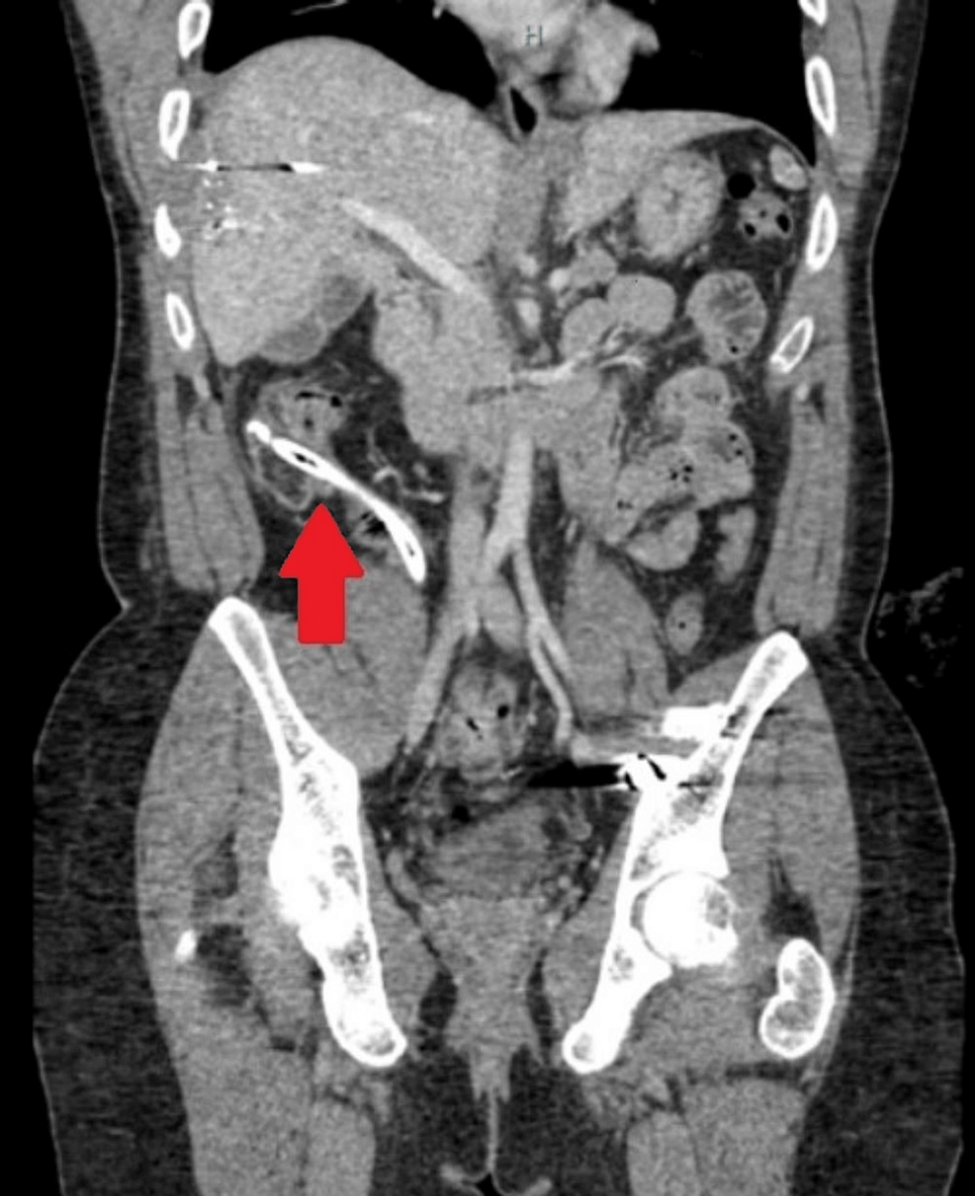
CT imaging with arrow indicating retained Malecot catheter

Informed consent for publication was obtained from the patient. The patient is a 24-year-old male who initially presented to the trauma bay of a level 1 trauma center after suffering gunshot wounds to his right lateral chest and the right lower quadrant of his abdomen with resulting hypotension and tachycardia. The patient was intubated in the field and a femoral central line and chest tube were placed in the trauma bay. He was taken immediately to the operating room for exploratory laparotomy. In a damage control approach, injuries to the diaphragm were repaired and the liver underwent perihepatic packing. An ileal enterectomy, right hemicolectomy, and rectosigmoidectomy were performed and the gastrointestinal tract was left in discontinuity in favor of a period of hemodynamic resuscitation. The left ureter was found to be transected on presentation and was thus ligated and reattached the following day. Post-injury day (ID) #1 a stapled functional end-to-end ileocolic anastomosis, descending colon end-colostomy with Hartman procedure and left ureteral reimplantation were performed. The patient’s hospital stay was prolonged and complex. On ID#5 he underwent thoracoscopic decortication for an empyema. On ID#9 the patient underwent angioembolization of a hepatic pseudoaneurysm to prevent the possibility of future hemorrhage. He returned to the operating room on ID#9, ID#11, and ID#13 for small bowel obstruction with adhesiolysis and wound dehiscence, serosal repairs, and eventual vicryl mesh closure. On ID#30, he developed a small bowel ECF. An Eakin Fistula and Wound Pouch wound management system (WMS) was placed to gravity drainage in order to control fistula drainage. He was discharged to a rehabilitation facility on ID#49. On ID#90 he was discharged home with the WMS for ECF management with plans for a future abdominal wall reconstruction, fistula takedown, restoration of bowel continuity, and potentially colostomy closure. On ID#104 he presented to clinic with excoriated skin and poorly controlled small bowel output. A mushroom-tipped 20-French Malecot catheter was placed in the proximal fistula to divert drainage into the WMS. The mushroom-tip portion was introduced at the proximal end of the fistula at the maximum length that would allow the distal portion of the catheter to be placed inside the drainage section of the WMS. Home health nursing services assisted him with his WMS. On ID#113, the patient returned to the emergency room with abdominal pain and reported that the Malecot catheter had disappeared and was no longer externally visible. The patient was certain that the Malecot had not been removed and unintentionally not replaced during his WMS dressing change with the home health wound care nurse. CT imaging revealed the 30 cm mushroom-tipped Malecot in the distal limb of the fistula traversing the ileocolic anastomosis (Figure 1).

We postulate that either the home health care services or the patient himself unintentionally replaced the 20F Malecot in the distal rather than the proximal limb of the fistula during a dressing change, but we are unable to definitively explain how the catheter migrated to the locations noted on CT scan.

**Figure 2:**
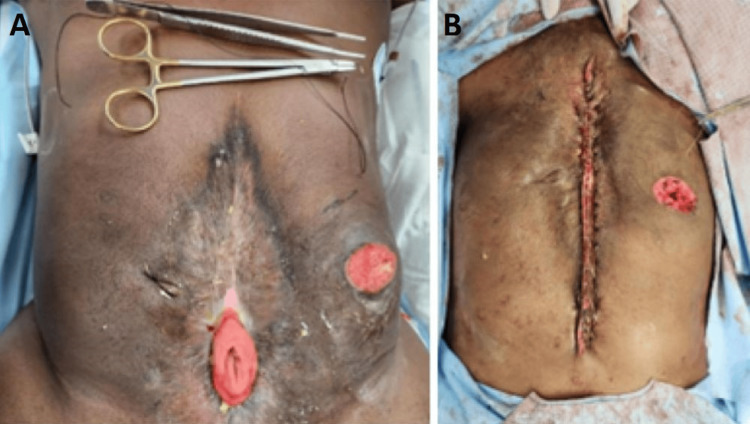
Surgical takedown of the enterocutaneous fistula (A) Pre-operative photo of the patient's enterocutaneous fistula. (B) Post-operative photo from the patient's fistula takedown.

Management options were discussed including aggressive bowel stimulation, endoscopy via the colostomy, as well as an open-operative approach. Due to his frozen abdomen and potential additional small bowel loss or harm at this stage of the fistula maturity, non-operative management with bowel stimulation, consisting of polyethylene gycol 17 grams twice daily, senna 17.2 milligrams twice daily, and saline enemas, was initiated. The patient was able to eat, and he had fistula output without obstructive symptoms. The patient was discharged home on ID#118 on laxatives and the catheter passed spontaneously through the colostomy without further invasive procedures two days thereafter. On ID#352, approximately one year after his initial injury, the patient underwent fistula takedown and small bowel anastomosis (Figures 2A, 2B).

The abdominal wall was not completely closed at this time. Repair of the resultant hernia will be considered at a future date if the patient is willing and continues to recover well. The patient is currently able to maintain adequate nutritional and lower bowel function.

## Discussion

Management of retained foreign bodies is guided by the existing literature and is a matter of clinical concern in emergency medicine, gastroenterology, and general surgery. While foreign bodies are most commonly present as retained surgical instruments, they can also present within the gastrointestinal tract via ingestion or anal insertion, with the majority of these objects passing spontaneously without the need for any operative intervention [6]. This is particularly common in pediatric populations, as ingestion of inappropriate objects is more common in this demographic and thus much of the existing literature regarding gastrointestinal retained foreign objects focuses on this population as well [5]. Robust studies regarding foreign body management in adults are rare.

The management of retained foreign bodies within the gastrointestinal tract is dictated by the characteristics of the foreign body as well as the anatomical location. Elongated, defined as greater than 6cm in length, or sharp-tipped objects are considered a greater risk for perforation and further migration and thus are typically removed, with endoscopy being the most common option for removal [4]. While existing literature indicates that most foreign bodies retained within the gastrointestinal tract will pass spontaneously without the need for any operative intervention, there does remain a risk of impaction and obstruction [6]. This typically occurs at the angles of the intestine or areas of narrowing, such as the duodenojejunal junction or the terminal ileum [7]. Existing guidelines thus take anatomical location into account; for example, elongated objects above the proximal duodenum are recommended for urgent endoscopic removal [8].

When intervention is indicated, endoscopy is the preferred approach for removing foreign bodies retained within the gastrointestinal tract and has a success rate of greater than 95% at removing the object [6]. Gastroenterological literature recommends urgent (within 24 hours) invasive management for sharp, corrosive, or elongated foreign bodies exceeding 6cm in length [7,9]. This recommendation is of particular relevance to our case, as the retained Malecot catheter was an elongated tube that was 30cm in length. An additional concern in this unusual case is that the tube contained within a functional end-to-end ileocolic anastomosis at the time and therefore of even greater concern for both capacity to pass completely and for perforation or obstruction in a hostile abdominal environment. Existing literature would recommend operative or endoscopic removal rather than expectant management if the literature was interpreted strictly without regard to the unique clinical circumstances. This case is, to our knowledge, the only reported case of a 30cm Malecot mushroom-tipped catheter within a functional end-to-end anastomosis in a penetrating abdominal injury with resultant entero-atmospheric fistula and a colostomy. Therefore, with little existing prior guidance, this foreign body’s location and length posed clinical concerns that led to our decision to pursue initial expectant management. Most existing guidelines are based on the management of orally ingested objects in pediatric populations and are of little value as a guide. Additionally, the patient had a frozen abdomen due to his extensive operative history, influencing the gastroenterologist’s decision for endoscopic intervention. This case does not fall neatly into pre-existing guidelines and presents a unique insight into a very rare presentation of a retained foreign body in the gastrointestinal tract. Thus, our case highlights an area in which the existing literature and management guidelines may not apply.

## Conclusions

Since the advent of the damage control approach to both trauma and complex general surgery, survivorship after extensive surgical intervention, complicated postoperative courses, and the development of complex abdominal wall challenges and fistula formation are no longer a rare entity. Additionally, foreign body retention, ingestion, and insertions and their management are familiar encounters to surgical specialists.

Our case demonstrated a Malecot catheter of 30cm in length inadvertently introduced into the distal limb of an entero-atmospheric fistula completing passage of the remaining gastrointestinal tract via a stapled functional end-to-end anastomosis and exiting a descending colostomy, fortunately without the need for either endoscopy or open surgery in a young male with a hostile, frozen abdomen. This unusual case demonstrates that long, blunt retained objects in the gastrointestinal tract may pass without complication. It should be noted that the patient had an absence of the ileocecal valve. In such cases, clinicians should exercise their best clinical judgment and may initially consider expectant management rather than operative intervention. We are hopeful our experience may assist others in decision-making should cases with similar clinical characteristics present.
